# A population-based cross-sectional study examining homicides among community-dwelling older adults in Victoria, Australia: A study protocol

**DOI:** 10.1371/journal.pone.0292837

**Published:** 2023-10-13

**Authors:** Briohny Kennedy, Lyndal Bugeja, Jake Olivier, Sjaan Koppel, Jeremy Dwyer, Joseph Ibrahim

**Affiliations:** 1 Department of Forensic Medicine, Monash University, Southbank, Victoria, Australia; 2 School of Mathematics and Statistics, University of New South Wales, Sydney, New South Wales, Australia; 3 Monash University Accident Research Centre, Monash University, Clayton, Victoria, Australia; 4 Coroners Prevention Unit, Coroners Court of Victoria, Southbank, Victoria, Australia; Yale University, UNITED STATES

## Abstract

**Background:**

There is a need for both descriptive and analytical evidence on the factors associated with older adult homicide. The current landscape is insufficient because most published research is confined to the United States, and contains insufficient data about the homicide context. This study protocol describes the proposed method for examining the characteristics and factors associated with older adult homicide in the Australian state of Victoria, using data generated for the criminal and coronial investigation into these deaths stored in the Victorian Homicide Register (VHR). Outcomes will support practitioners, policy makers and other key stakeholders to strengthen prevention strategies to reduce the risk of future homicides among older Victorians.

**Methods:**

This study will comprise a single-jurisdiction population-based cross-sectional design to analyse consecutive cases of homicide among community-dwelling older adults in Victoria, Australia for the period 2001 to 2015. All homicides of adults aged 18 years and older, and where the Coroner’s investigation is completed at data extraction will be included. Variables will be selected in accordance with elements of the social-ecological model (i.e., individual, interpersonal, incident, and community). This will include: socio-demographic characteristics; presence of mental or physical illness; deceased-offender relationship; nature of any abuse between the deceased and offender; incident location and weapon used; the presence of alcohol or drugs; and criminal justice outcomes. Homicide rates per 100,000 population will be calculated for older adults (aged 65 years and older) and younger adults (aged 18–64 years), and compared as rate ratios using Poisson regression. Descriptive statistics and cross-tabulation will be generated for factors associated with homicide for older compared to younger adults. Homicide typologies based on deceased-offender relationship and motive will be explored within group and family homicides will be compared between older and younger adults.

## 1. Introduction

### 1.1. Background

Substantial growth in the proportion of community-dwelling older adults has been observed globally as a result of lower birth rates and increased longevity [1]. As the ‘baby boomer’ generation matures, the proportion of adults aged 65 years and older is expected to double between 2019 and 2050 [1]. In Australia, the proportion of the population aged 65 years and older, which was 16% in 2020 [2], is expected to reach 19% by 2050 [3], and those aged 85 years and older (2% in 2020) [2] are expected to reach four percent [3].

While it is not certain whether the increase in the number of adults over 65 years old in Victoria, Australia will equate to an actual increase in homicide incidence, there is little to indicate these numbers will decrease. One reason for this is that despite trends for decline in the general homicide rate, the rate for older adults has changed little over time [4]. In fact, trends in Australia for older adults have been reported as either slightly increasing [5] or stable [6], rather than decreasing, which has also been observed more recently in the United States [7,8]. This suggests a likely increase in older adult homicide events even without a notable change in rate. Adding to this, the most recent report on homicide in Australia noted a 16% increase in homicide on the previous year and the highest since the 2005–06 reporting period [9]. Contemporary issues that may contribute to a future with an increase in older adult homicides include inter-generational familial stressors, such as financial issues, caregiver stress, and increasing mental illness in the community [10].

With this increase in the older population, it is expected that there will be a concomitant increase in the frequency of older adult homicide. Research that specifically investigates the factors contributing to homicide among older adults is limited [4,10,11] despite an increasing volume of research describing the prevalence, characteristics and prevention of elder abuse and neglect [12–14]. According to the Global Status Report on Violence Prevention [15], key global data on homicide lacks the specificity required for research into this age group.

The first meta-analysis of international older adult homicide research described a pooled prevalence of older adult homicide of 2.02 per 100,000 population, and that older adult homicide victims were predominantly killed in their home, by either a family member or a person that was a stranger to them, and during either an argument or a criminal activity against them [4]. Compared to younger adult victims, older victims were significantly more likely to be female, die at the home location, by an offender that was a stranger to them, and during a criminal activity against them. Conversely, older adults were significantly less likely to be killed during an argument, or with the use of a firearm [4]. A recent review by Rogers and Storey [11] identified an emerging typology of the older female victim killed by someone known to her in her own home.

These contemporary reviews highlight the need for further evidence on the distribution and determinants of homicide amongst community-dwelling older adults. Furthering this research domain requires a deeper understanding of factors associated with the fatal incident that are potentially preventable. These include whether homicide results from ongoing abuse or neglect, an understudied area [11], and whether there were individual factors related to deceased or the offender such as presence of alcohol and/or other drugs, psychological and medical history and other sociodemographic variables [10,11,13,16].

The nature of adult homicide is heterogeneous and typologies are mostly characterised by the deceased-offender relationship [17,18], with sub-types commonly derived from the homicide motive [11,17,18]. Older adult homicides predominantly occur in the process of criminal activity or during an argument [4,11,19,20], though further data on the individuals involved and the homicide incident are lacking. This affects our understanding of what contributed to the event and how it might have been prevented. Offenders of older adult homicide are frequently known to the older adult victim [4], and familial offenders are not necessarily intimate partners [10]; for example their adult children [21], of which motives and contributing interpersonal factors are not well documented [10,22]. While much work has been done to specify intimate partner homicide (IPH) typologies [23,24], research into older adult IPH has done little more beyond the categorisation of ‘mercy killing’ [25], and deserves greater attention. Importantly, while older adult homicide victims are frequently killed by someone known to them [4,11], the proportion that are killed by their intimate partner is small when compared with younger adults [26,27].

### 1.2. The social-ecological model and older adult homicide

Violence is recognised as a complex social and public health problem [28]. Public health conceptual frameworks have been increasingly applied in interpersonal violence research and policy to identify and evaluate evidence-based primary, secondary and tertiary prevention strategies [29]. One such conceptual framework, the public health approach states that once a problem is defined, risk and protective factors are then identified so that appropriate prevention efforts can be developed, assessed for effectiveness and implemented [30].

**Fig 1:**
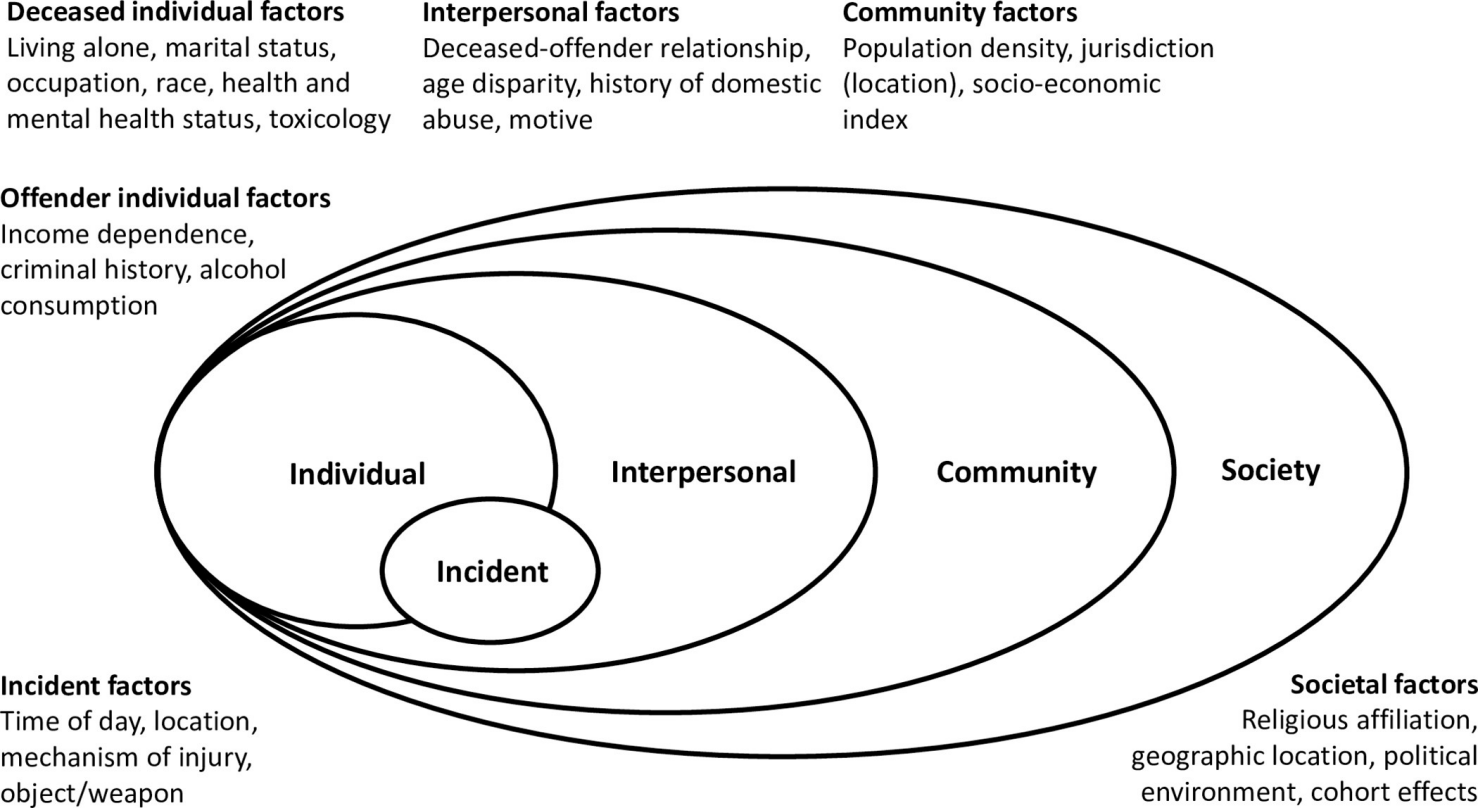
Modified social-ecological model–older adult homicide.

Risk and protective factors can be examined using another public health framework, the social-ecological model (SEM), first introduced by Bronfenbrenner [31]. As with the public health approach, the SEM has been applied to strengthen the understanding of factors associated with and protective against interpersonal violence [18,30–32]. While Bronfenbrenner depicted the nested ecological model components as micro-, meso-, exo- and macrosystems, public health practitioners, and more specifically violence prevention researchers, have reframed the nested categories as individual, interpersonal, community, and societal [30,33]. In a modification appropriate to better understanding homicide, the incident location, mechanism of injury, weapon, injury severity, and time of day can be presented as a stand-alone ‘incident’ category that intersects the individual and interpersonal categories (Fig 1).

Individual level factors known to be associated with homicide among older adults include: sex; race and social isolation for the deceased; mental and physical health, and historical exposure to violence of both the offender and the deceased; historical drug and alcohol abuse or presence of alcohol in the offender at the time of the homicide event; and previous contact with the legal system or suicide threats by the offender [10,11,21]. In their study of older adult homicide-suicide, Malphurs found offenders had a low documented history of receiving psychiatric care (5%), which contrasted to the high proportion reported as displaying mental illness (51.9%) [34].

Across adults of all ages, mental illness, history of substance abuse [35], and a criminal record for intimate partner violence (IPV) may increase a person’s chance of committing homicide [36]. Previous criminal history, however, is not always present in IPH [37], and a history of criminal charges does not definitively indicate that a homicide will take place [38]. Being a migrant or an ethnic minority is also associated with a higher likelihood of IPH victimisation [39,40].

At the interpersonal level, factors associated with older adult homicide include: the deceased-offender relationship; motive; financial dependence of the offender on the deceased; physical dependence of the deceased on the offender; and for intimate partners, recent or impending separation; history of domestic violence/discord; and the age difference between the deceased and offender [10,22,27]. Malphurs [34] found homicide-suicide perpetrators were three times more likely to be a caregiver than those who died by suicide alone. The study also identified that spouses, followed by other family members, were the primary offenders of homicide-suicide, with physical or verbal marital discord noted for 19% of incidents, and 29% of homicide-suicides being related to an impending divorce.

It is not known whether established risk factors for IPH in adults in general, such as recent separation, access to weapons, a history of actual or threatened violence [35,39], or pre-migration trauma [39], apply to the same extent in older adult homicide. Being married rather than cohabiting was an identified protective factor for IPH across adult age groups [27].

A recent bereavement or change in life circumstance in the deceased has also been noted in an analysis of domestic homicide reviews in the UK [10], as well as dependence on the deceased by the offender and a caring role within the relationship dyad [41].

Incident level factors include: weapon used; location and time of day of incident; and the severity of injury, for example the use of excessive force [42]. A recent meta-analysis of older adult homicide found firearms to be used significantly less in older adults compared to younger adult homicide victims, and the homicide location to be significantly more often at home [4]. The mechanism is most frequently by sharp object, firearm or blunt trauma [11] and differs significantly to younger adult homicide [4]. In addition to the established concept that older adults are more susceptible to injury and a poorer recovery from physical assault [43], are studies concluding that attacks on older adults can involve a use of excessive force [42,43]. Additional empirical evidence is required to confirm excessive force is consistently more present than in younger counterparts.

Community level factors include geographic area and socio-economic variables such as household structure [44]. Older adult homicide rates vary by geographic location, for example prevalence is higher in Miami, Florida, compared to Chicago or Houston [26], and is higher for the US overall compared to other high-income Organisation for Economic Co-operation and Development countries [45]. In their analysis of culture and context, Weaver and colleagues [44] found that lower religious adherence and higher robbery rates at the community level were associated with higher prevalence of older adult homicide [44]. Adult IPV victims residing in rural locations have also been identified to have greater risk of homicide [39,46]. Other frequently examined community level factors in the US in particular, are the association of gun ownership and relevant state-based legislation on fatal violence [45,47].

Societal level factors include governmental and other cultural influences [48]. Exploring these societal structures is beyond the scope of the current study. Studies directed at the societal or structural level usually involve comparing large geographic areas according to the prevalence of homicide or another outcome of interest with known or potential factors at that level, focussing less on the individual, interpersonal, incident and community level factors [13,44,48].

Through the lens of the modified social-ecological model, there are still relevant details at the individual-, interpersonal- and incident-level that have not been described for older adults. Specific areas that require a greater understanding include potential risk factors for offenders [4,11], for example history of violence or mental illness or contact with the criminal justice system. This information is seldom reported in older adult homicide research, with the exception of research describing offender-only data collected via the criminal justice system [16].

Data that provides information about the interaction between the deceased older adult and the offender is also crucial, but is not usually explained beyond the relationship [49,50]. While understanding if there was a history of interpersonal violence is well recognised in intimate partner violence research [51], it is not frequently available in older adult homicide research. The same is true for history of recent separation, and age difference between the deceased and offender in partner homicide.

The incident circumstances also require a greater than basic description of weapon, location and motive [43,50], for example the use of alcohol and other drugs proximal to the event.

### 1.3. Medico-legal death investigation

In Australia, as in many other developed countries, unexpected and unnatural deaths, including suspected homicides, are required to be reported to a Coroner. Following a criminal investigation, the Coroner may perform additional investigations before they make a finding on: the deceased’s identity, cause of death and, in some cases, relevant proximate and historical circumstances leading up to the death [52]. This routinely collected information is recognised as both a reliable and valuable source for research [53].

Studies that have relied on administrative databases with uniformly collected data tend to provide a limited level of detail [43]. In the area of domestic homicide, there have been some promising studies from the United Kingdom containing a greater level of granularity [10,21], though these are derived from text-based documents that are usually qualitative in presentation and not prepared in a uniform manner.

Many studies examining homicide, particularly from offender-based data only, include cases where an offender has been identified and successfully prosecuted [16,54]. The use of coronial data permits inclusion of cases regardless of whether an offender was identified or there was a conviction.

In addition, domestic or family violence death reviews have been established in many Australian jurisdictions [52,55], and have led to the development of data systems to support their work. The first of these data systems, the Victorian Homicide Register (VHR), was established at the Coroners Court of Victoria (CCOV) by the Coroners Prevention Unit in 2014 to support family violence death reviews as part of their Victorian Systemic Review of Family Violence Deaths [52]. Coded and free text information is recorded for both the deceased and offender(s) and includes: socio-demographic characteristics; deceased-offender relationship; physical and mental health history (including alcohol and other drug use); criminal history; service contact history; and interpersonal factors, including any history of domestic violence perpetration or victimisation. This detailed and rich data makes the VHR a unique and useful information source to examine homicide at the individual, interpersonal, incident and community levels as set out by the modified SEM (Table 1).

**Table 1:** Individual, interpersonal, incident and community level variables associated with adult homicide and availability in the Victorian Homicide Register.

	Variable type	Empirical evidence	Available in VHR, deceased only (D), offender only (O) or both (B)	Older cases (n = 63) n (%)		Younger cases (n = 695) n (%)	
**Individual level**		** **					
**Accommodation type**	Nominal	Residential instability was associated with homicide and suicide in Australia, Canada and the United States [56]. Financial stressors, including housing need was noted in 29% of perpetrators and 31% of victims of AFH [41].	**B**	42	66.7	132	19.0
**Age in years**	Interval	Over 80% of perpetrators of older adult IPH offenders were also older adults [21]. Oldest old homicide victims (aged 85 years and over) were more frequently female, killed by a family member and killed with a personal contact weapon; Young old (65–74 years) were more frequently killed with knives in argument-related circumstances [57]. Abuse risk increases with age [22].	**B**	63	100.0	695	100.0
**Assessed physical disability**	Binary	Adults with disability at higher risk of violence and people with intellectual impairments 1.6 times more likely to have experienced violence than those that have not [58]. A larger proportion of offenders of elderly homicide had a below average IQ (13.4%) than other adult homicide offenders [16].	**B**	63	100.0	176	25.3
**Cultural and linguistic diversity** ^**a**^	Binary	Older adult homicide victims predominantly white or more likely to be white when compared with younger adult victims for several studies [11]. Perpetrators of intimate partner homicide-suicide (H-S) more likely to be white [59]. Perpetrators of intimate partner femicide more likely to be African American or Hispanic [59]. Being foreign-born increases the likelihood of being killed by a partner than by another relationship type [39]. Older adult homicide victims more likely to be white than younger victims [57,60]. White homicide victims were at four times the odds of being in a H-S event [61]. Victims of intimate partner homicide were twice as likely, and their offenders three times more likely, to be black compared with non-fatal IPV victims and offenders [40]. Compared with homicide victims in general, IPH victims were 1.82 times more likely to be foreign-born [40]. In Australia, 20% of homicide victims were born overseas for 2018–19 [62].	**B**	42	66.7	325	46.7
**Diagnosed or suspected mental illness** ^**b**^	Nominal	Psychosis more frequent in elder homicide offenders, compared with offenders of younger adult homicide [16]. Mental disorder of the offender significantly greater for older adult homicides (16.8%) than for other homicides (3.8%) [5]. More than half (51.9%) of perpetrators of older adult H-S had psychiatric symptoms [34]. Mental health difficulties were present in 78% of adult family homicide (AFH) perpetrators, which was significantly greater than for victims (28%) [41]. In Australia, 10–15% of homicide offenders have a known mental illness (including depression) [62]. Prior mental health problems, including depression and personality disorder in the perpetrator a risk factor for IPH of females [59]. IPH offenders were 1.64 times more likely to suffer a psychiatric disorder than other homicide offenders [40]. Persons with mental illness 3.86 times more likely to have experienced violence than those without mental illness [58]. Depression a risk factor for elder abuse victimisation [22].	**B**	53	84.1	166	23.9
**Employment status**	Nominal	Erratic employment patterns were observed in 75% of elderly homicide offenders [16]. Where employment was identified, 70% of AFH perpetrators were unemployed and 45.5 were unemployed long-term, double the proportion for victims [41]. Perpetrator unemployment increased the risk of female IPH by 4.42 times [59]. IPH offenders were 2.32 times more likely to be employed than other homicide offenders [40].	**B**	58	92.1	194	27.9
**Family history mental illness**	Binary	Family substance-related problems identified in 58% of elder homicide perpetrators [16]. Alcohol abuse history identified in parents of AFH perpetrators [10].	**B**	63	100.0	176	25.3
**Historical exposure to violence**	Nominal	AFH perpetrators were significantly more likely than their victims to have experienced childhood trauma (51.5%) or childhood abuse (45.5%) [41]. Adult child perpetrators of adult family violence can have a history of childhood exposure to violence, abuse and neglect [10]. Pre-migration trauma associated with higher levels of domestic homicide risk factors [63].	**B**	61	96.8	178	25.6
**History of substance abuse**	Nominal	Alcohol misuse was found in 62% and substance misuse in 60% of AFH perpetrators, both significantly greater than for victims [41]. Alcohol abuse a risk factor for IP femicide [59]. Older adults with substance abuse more susceptible to abuse by others, and alcoholism associated with family violence perpetration against older adults [22].	**B**	60	95.2	151	21.7
**Indigenous status**	Nominal	Older homicide victims in Australia were significantly less likely to have been identified as aboriginal or Torres Strait Islander (2.4%) when compared with other homicide victims (13.4%) [5]. Indigenous men and women in the US are at increased risk of homicide [39]. Indigenous Australians over-represented as homicide victims and offenders [6]. Indigenous Australians comprised 13% of all homicide victims and 16.5% of offenders; The indigenous homicide rate was 3.54 per 100,000 for 2018–19 [62].	**B**	36	57.1	184	26.5
**LGBTI+**	Nominal	Threats to harm children a risk factor for intimate partner femicide by a same-sex partner [59]. LGB persons experience IPV at the same or increased levels as heterosexual adults, and there is some evidence that IPV incidence is higher for transgender persons [39].	**B**	60	95.2	169	24.3
**Mental health treatment** ^**c**^	Binary	Offenders of elder homicide had more frequently received prior psychiatric treatment than offenders of younger adult homicide [16]. Just 5% of elder H-S perpetrators were receiving psychiatric care, less than for suicide only [34].	**B**	63	100.0	176	25.3
**Pain chronic acute cancer**	Binary	Physical health problems were significantly greater in victims (59.1%) than for perpetrators (31.8%) of AFH [41].	**B**	63	100.0	176	25.3
**Physical illness present** ^**d**^	Binary	Victims and perpetrators of AFH may have physical illness requiring care [10]. Partners of 11% of H-S perpetrators were in nursing homes versus 0% for suicide alone [34]. Heart disease in higher prevalence than population in elder H-S perpetrators at 85% [34]. Having a long-term physical or other health problem increases likelihood of abuse [64].	**B**	63	100.0	176	25.3
**Physical injury present**	Binary	(See physical illness present)	**B**	63	100.0	176	25.3
**Prior offending**	Nominal	Offenders of elder homicide less frequently had a prior violent conviction, than offenders of homicide in younger adults [16]. There was a history of criminal offences in most AFH perpetrators (71.2%) and some victims (5.8%) [41]. Almost half of AFH offenders had a history of domestic abuse, particularly IPV [41].	**B**	56	88.9	148	21.3
**Prior suicide attempt**	Nominal	Offenders of adult IPH were 1.62 times more likely to have had prior suicide ideation or attempts than offenders of non-fatal IPV [40].	**B**	60	95.2	117	16.8
**Prior suicide ideation**	Nominal	Older adult H-S perpetrators five times less likely to have threatened suicide than in suicide alone, and less likely than suicide alone to have had suicide ideation prior [34]. Threats of suicide a risk factor for partner H-S of adult female victims [59].	**B**	60	95.2	125	18
**Receiving current medical treatment**	Binary	Of the AFH perpetrators with mental health issues, 86.5% were also receiving support for physical health issues [41]. Healthcare service contacts were the most prominent contacts made individually by IPH perpetrators and victims [65].	**B**	63	100.0	176	25.3
**Recent and other service contacts** ^**e**^	Binary	A criminal justice history present in 71.2% of AFH perpetrators, significantly greater than for victims (25.8%), and police contact noted in 75.8% of perpetrators [41]. Service contacts made with the justice system or health system for over 75% of deceased and offenders in IPH; the majority of contact were made within a month of the homicide, and more service contacts were made by offenders than the deceased [65].	**B**	63	100.0	176	25.3
**Sex**	Binary	Males victims more frequent than females [57,60], pooled estimate 46.3%;[4] Females more frequent compared to younger adult victims [57,60], pooled estimate: OR = 2.5 (95% CI = 2.02–3.10) [4]. Victims of adult homicide by a caregiver were predominantly female (63.2%) [66]. Male eldercide victims more likely to be killed by someone aged under 45 years, and to be killed by a stranger, while females more likely to be killed by someone over 45 years and by a spouse or a child [67]. Case series of fatal elder abuse in Japan identified predominantly female victims (67%) [68]. Elderly H-S victims predominantly female; being an elderly male homicide victim reduced the odds of becoming a H-S victim [61]. Offenders of older adult homicide predominantly male, but female offenders more prominent compared to younger adult homicide [11]. Adult females are 9 times more likely to be killed by an intimate partner than males [59].	**B**	63	100.0	695	100.0
**Interpersonal level**							
**Bystander of family violence within dyad**	Binary	(See historical exposure to violence.)	**B**	63	100.0	695	100.0
**Deceased relationship to offender** ^**f**^	Nominal	Pooled estimate for stranger offender 24.2% [4]. Offender more often a stranger than in younger homicides [16,69,70](Pooled OR = 1.81 [95% CI 1.66–1.98], p < .001);[4] Pooled estimate for familial offender: 25.2% [4]. Offenders reported as family members less consistently, including: more frequently than in younger adult homicides [57,69,70], or with little difference in proportion (OR = 0.97, 95% CI = 0.89–1.06, p = 0.4961 [4]. Older adults less frequently killed by spouse than younger adults [16,27]. Older adult H-S predominantly carried out by spouses, followed by children [34,61]. Assaults on older adults by family members and people known to them are associated with higher fatality risk than assaults by strangers [49]. Adult homicides committed by a caregiver were predominantly carried out by sons (41.2%) or daughters (29.4%) [66]. Fatal elder abuse and neglect in Japan observed to be frequently by family members, i.e. adult children or in-laws, grandchildren [68].	**D**	60	95.2	670	96.4
**History of family violence in dyad**	Nominal	Elder H-S featured a history of domestic violence (DV) 25% of the time compared to 5% of suicide only deaths [34]. IPH preceded by DV against female partner in 67–75% of cases [59].	**D**	61	96.8	391	56.2
**Incident classified a family violence homicide**	Nominal	AFH recognised as having different characteristics to other adult and older adult homicides [4,11,41].	**D**	61	96.8	417	60.0
**Intimate partner relationship duration**	Nominal	Long-standing relationship a risk factor for IP femicide [59].	**D**	31	49.2	164	23.6
**Intimate partner relationship status**	Nominal	Marriage identified as protective of IPH, being older identified as protective of IPH [27]. Being married may increase exposure to elder abuse [22].	**D**	33	52.4	240	34.5
**Motive** ^**l**^	free text	Pooled estimate: motive argument 36.1% for older adult homicides [4]. Less frequent than for younger adult homicides [16,69,70], Pooled OR = 0.33, 95% CI = 0.28–0.39, p < .001) [4]. Pooled estimate: motive felony 30.8% for older adult homicides [4]. Felony motive greater for older victims than younger victims; [16,57,69,70] Pooled OR = 2.78, 95% CI = 2.58–2.99) [4]. Older adults at higher risk of dying from an assault than younger victims and assaults on older adults by family members and people known to them are associated with higher fatality risk than assaults by strangers [49]. A common H-S typology involves older adults where the victim was suffering from a neurological disorder and offender also sometimes experiencing a decline in health [71].	**O**	50	81.0	207	25.9
**Other violence and threats by perpetrator** ^**g**^	Binary	Perpetrators of AFH had a criminal history of DV offending in 48.5% of cases [41]. Threats to harm children a risk factor for intimate partner femicide by a same-sex partner [59].	**B**	63	100.0	176	25.3
**Perpetrator abused or threatened victim** ^**h**^	Binary	Nonfatal strangulation, prior choking, forced sex, stalking, threats to kill, threats with a weapon and IPV during pregnancy identified as risk factors for IP femicide [59]. (See also history of family violence in the dyad)	**B**	63	100.0	176	25.3
**Perpetrator attitude of violence towards women, children, elderly**	Binary	Attitudes that justify the use of violence moderately associated with IPV risk [72]. Patriarchal beliefs a risk marker for both intimate terrorism and IPV [73]. (See also perpetrator abused or threatened victim and Other violence and threats by perpetrator)	**B**	63	100.0	176	25.3
**Perpetrator breached current intervention order**	Binary	There were current intervention orders for 11% of IPH victims [74].	**B**	63	100.0	176	25.3
**Perpetrator breached previous intervention order**	Binary	Around one quarter of intervention orders imposed between 2004 and 2007 were breached [75].	**B**	63	100.0	176	25.3
**Perpetrator had access to weapons**	Binary	Pooled OR of 2.0 (CI = 1.56–3.02) for homicides where victims had firearms in the home compared to those that did not [76]. Gun accessibility, availability and ownership risk factors for adult femicide [59].	**B**	63	100.0	176	25.3
**Perpetrator history of family violence**	Binary	AFH perpetrators had a criminal history of DV offending in 48.5% of cases, in particular for IPV [41]. Compared with non-fatal IPV offenders, IPH offenders were almost twice as likely to have a history of violence in earlier relationships [40].	**B**	63	100.0	176	25.3
**Perpetrator of family violence within dyad**	Binary	(See History of family violence within the dyad.)	**B**	63	100.0	176	25.3
**Recent or impending separation** ^**i**^	Nominal	Impending divorce a primary cause in 29% of H-S of older adults [34]. Estrangement a risk factor for intimate partner femicide [59]. Divorce rate a predictor of homicide at the structural level [48].	**D**	61	96.8	362	52.1
**Victim of abuse** ^**i**^	Binary	Presence of emotional abuse of deceased identified in 5/15, financial abuse in 3/15 fatal elder abuse and physical abuse identified in 13/15 fatal elder abuse cases in Japan [68]. Elder abuse was present in 13.6% male and 22% female victims of older adult homicide [77]. Increased all-cause and specific mortality (physical and emotional abuse) [78,79].	**B**	63	100.0	176	25.3
**Victim of family violence**	Binary	History of domestic abuse common in older adult IPH and parricide [21]. (See also History of family violence within the dyad.)	**B**	63	100.0	176	25.3
**Victim vulnerable to perpetrator** ^**k**^	Binary	Perpetrator of H-S in older adults was a caregiver to deceased in 31.6%-42% of cases, three times more likely to be a caregiver than for suicide alone [34]. Financial difficulties were present for 31.8% of victims and 30.3% of perpetrators, and a caring role in the relationship was identified for 21.2% victims and 24.2% of AFH perpetrators [41]. The deceased was a carer for, or was being cared for by, the perpetrator in 60% of older adult domestic homicides [10]. Almost half of adult victims of homicide by a caregiver were over 80 years old [66]. Both dependence of a victim on a perpetrator and dependence of perpetrator on victim associated with elder abuse by adult children [22].	**B**	63	100.0	176	25.3
**Incident level**							
**Coroners finding**	Nominal	NA—administrative	**D**	63	100.0	695	100.0
**Date of death**	Interval	NA—administrative	**D**	63	100.0	695	100.0
**Date of incident**	Interval	NA—administrative	**B**	63	100.0	695	100.0
**Deceased consumed alcohol and drugs prior to incident**	Nominal	Significantly less older homicide victims were tested for alcohol and illicit drugs;[60] Significantly less older homicide victims tested positive for alcohol and illicit drugs [60]. There was a 1.85 higher likelihood of IPH where the victim was under the influence of alcohol at the time of the incident [40].	**D**	63	100.0	695	100.0
**Incident location**	Nominal	Pooled estimate for location home: 71.4%;[4] Older adult victims more likely to be killed in their home than younger adults [16,57], at more than three times the likelihood: OR = 3.87 (95% CI = 3.45–4.35) [4].	**D**	61	96.8	276	39.7
**Justice outcome**	Nominal	Significantly more 2nd degree murder convictions for offenders of elder homicide and no difference in guilty plea between offenders of elderly versus non-elderly homicide [16].	**O**	60	96.8	517	64.8
**Mechanism**	Nominal	Pooled estimate frequency of firearm mechanism: 24.5%;[4] Older persons less likely to have been killed with a firearm than younger adult homicide victims [16,57,60,70], OR = 0.38, 95% CI = 0.36–0.40);[4] More frequently killed with beating, cutting, and strangling than younger victims;[60] Significantly larger proportion of oldest old (85 years and over) killed using personal weapons (i.e. knives) compared to younger adults [57]. Causes of death predominantly from hands and blunt-force mechanisms, i.e. subdural haemorrhage, strangulation, suffocation, observed in elder abuse fatalities in Japan [68]. Elder homicides involving firearms 7–10 times more likely to be H-S [61].	**D**	63	100.0	695	100.0
**Offender charged** ^**l**^	Nominal	Homicide clearance rate a predictor of homicide at the structural level [48]. (See also Justice outcome)	**O**	61	98.0	756	94.7
**Offender consumed alcohol and drugs prior to inciden** ^**l**^	Nominal	Offender consumed alcohol at 60% and used street drugs at 19% of elderly homicide offences, though not significantly different to younger homicides [16]. Perpetrators of AFH misused alcohol and other substances in 62.1% and 60.6% of cases, respectively [41]. For perpetrators of older adult H-S, 15% had BAC over 0.05, and 25% had analgesics detected in system, but no difference found to suicide alone [34].	**O**	62	100.0	798	100.0
**Offender sentenced** ^**l**^	Nominal	(See Justice outcome)	**O**	60	97.0	722	90.5
**Overkill**	Nominal	Moderate to severe injury was significantly more frequent in older adult homicides compared to younger adults [16]. Elderly female victims had a higher mean homicide injury score than younger adult victims, indicating excessive use of force in the older group [42].	**D**	63	100.0	695	100.0
**Single or multiple fatality**	Nominal	Elderly females over-represented in H-S in the elderly (81%) when compared to homicide in the elderly in general; Offenders of H-S in the elderly were predominantly intimate partners (72%), followed by children (10%) [61]. H-S and multiple victim incidents comprised 11.3% and 9.2% of older adult homicides, respectively [8]. Multiple family homicides (familicides) with victims of all ages can be distinctly grouped in terms of the perpetrator’s individual factors, motive and other situational factors [80].	**B**	63	100.0	695	100.0
Community level							
**Deceased’s local government area**	Nominal	Elder homicide in Northern Portugal predominantly occurred in rural settings [81]. Proportion of older adult homicide to younger adult homicide varied between major US cities, as did offender race, relationship and motive [26]. The elder homicide rate was higher in cities with above average homicide rates [26]. At the community level, education was the only factor found to be significantly associated with the overall elder homicide rate in Florida for 1976–85 [32]. IP femicide perpetrators more likely to be poor [59]. IPV victims in rural settings are at higher risk of IPH [39]. Community level resource (i.e. social security expenditure), GINI coefficient, distribution of wealth, age structure and other area level indicators of social structure are predictors of homicide rate [48].	**D**	63	100.0	545	78.4
**Incident local government area**	Nominal	(See Deceased’s local government area)	**D**	62	98.4	527	75.9

^a^Includes individual variables: Country of birth, Ancestry, Asylum seeker status, English proficiency, Mothers country of birth, occupation, Fathers country of birth, first language, main language, Refugee humanitarian visa, Religious affiliation, Residency status, Skilled worker visa, Spousal visa, Study visa, Time spent in detention, Visitor visa, Years lived in Australia, other visa.

^b^Includes individual variables: Behavioural syndromes associated with physiological disturbances, childhood or adolescent behavioural and emotional disorders, mental disorder due to substance use, mental retardation, mood affective disorder, neurotic, stress-related and somatoform disorder, organic mental disease, psychological development disorder, schizophrenia, schizotypal and delusional disorders, or unspecified mental illness.

^c^Includes individual variables: (Recent or other) Emergency department presentation for mental health, mental health treatment from a general practitioner, psychiatric treatment, psychological treatment, treatment as a voluntary or involuntary community patient, treatment as a voluntary or involuntary inpatient, treatment from a mental health practitioner, from community or outreach mental health services or from drug and alcohol services.

^d^Includes individual variables: Physical illness presence and other evidence of health issues.

^e^Includes individual variables: Corrections, courts, Department of Health and Human Services, drug and alcohol treatment service, emergency department, general practitioner, housing services, maternal and child health service, non-government services, other government services, police, sexual assault support services, specialist family violence service and engagement with Centrelink, Known to child protection services, Most recent CPS involvement, Prior child protection orders, active involvement from child protection services, current child protection order.

^f^Includes individual variables: Deceased relationship to offender and deceased-offender relationship a family homicide.

^g^Includes individual variables: Perpetrator assaulted non-family members, assaulted other family members, harmed or killed pets, physically harmed children, threat to non-family members, threat to other family members, threatened to harm or kill pets or threatened to harm children.

^h^Includes individual variables: Perpetrator choked victim, controlling towards victim, isolated or impeded victims’ freedom, physically assaulted victim, previously harmed victim with weapon, sexually assaulted victim, stalked victim, threatened to harm victim, threatened to kill victim, threatened victim with weapon, verbally abused victim or recent escalation of violence toward victim.

^i^Includes individual variables: Recent or impending separation, intimate partner intention to separate, and intimate partner separation duration.

^j^Includes individual variables: Emotional abuse, financial abuse, physical abuse, psychological abuse, sexual abuse and social abuse.

^k^Includes individual variables: Victim afraid of perpetrator, believed perpetrator could kill children or could kill them, dependant on perp financially, for care, for residential status, isolated by perpetrator, isolated for cultural reasons, recently given birth, unexplained injuries.

### 1.4. Comparison with younger adult homicides

Use of comparisons groups for exploring the factors associated with homicide in older adults is conceptually and logistically challenging. One approach is comparison with non-fatal and serious injury of older adults within the geographic region. The comparison of older non-fatal and fatal assault has been performed in the US [43,49,82], and these highlight substantive methodological issues. One of these issues is that data sources differ due to the original purpose of information collection. Specifically, data sourced from surveys or the healthcare system include different variables to data generated from the criminal justice process, with further disparity in variable definition and record-keeping reliability [43,57].

Another issue is that homicide is reported at a high level of accuracy compared to assaults which may be reported either to the police or measured through hospital presentations [83]. The latter may only represent a fraction of the actual number of assaults in the population for multiple reasons which are difficult to reconcile [49].

An alternative approach to comparison of older adult homicides is of populations in different geographic regions. This also problematic because of variance in methods for collecting data within each jurisdiction, and the inability to control for community-level factors that might influence the homicide rate. Examples include legislation, political environments or other socio-cultural factors [11,44].

Comparisons between older and younger adults is a commonly used data analysis method in the international homicide research literature [4,11,57]. It also facilitates hypothesis generation and is reasonable when the limitations are acknowledged. This approach also recognises that, an optimal comparison study population may not be available, not logistically feasible to obtain, or of a such a low frequency count to negate robust statistical analyses.

There are benefits to developing policy and prevention initiatives by determining whether the underlying factors for homicide between young and older populations are similar or different. At closer inspection, individual studies have identified that rather than being an intimate partner, the relationship is more likely to be intergenerational, for example parent-child or grandparent-grandchild [10,21] The recent meta-analysis reported that older homicide victims were significantly more frequently female, killed by a stranger in their own home and were less often killed with a firearm than their younger counterparts [4]. These established factors recognise older adults as a discrete group.

It is important to specify the factors unique to older adults as well as those shared with other age and sex cohorts in the population. In their evaluation of the state of older adult homicide research, Addington [50] identified the need to expand on the descriptive research, including increasing understanding of the diversity among victims, offender characteristics, in-depth analyses, and identifying risk and protective factors.

There is value in comparing older and younger adult homicides from within the same society in the Australian context. An increased recognition and response to family violence in Australia over the last decade has resulted in strategies focused on younger people (usually women with children) who experience violence from men they have been in an intimate or familial relationship with [84]. While the focus of this response is reasonable given the public health burden, the strategies may not address the factors and circumstances unique to older adults. Specific examples where the scope is not specific to older adults include firearm restrictions [85], and intimate partner homicide risk assessment tools [51].

### 1.5. Research questions

As outlined above, the VHR offers a rich and unique data source in which to provide a detailed description of the nature of homicide against the older adult. The modified social ecological model is a useful framework for examining these known and potential risk factors at the individual, interpersonal, incident and community levels (Table 1). A comparison between older adult and younger adult victims will also serve to contextualise the older adult homicide situation. The following research questions will be addressed: 1. What are the individual (victim and offender), interpersonal, incident and community level factors associated with older compared to younger adult homicide? 2. Are the key factors associated with homicide among older adults in Victoria the same as for younger adults? 3. What are the characteristics of older adult homicide typologies based on the deceased-offender relationship? 4. Are the key factors associated with homicide among older adults in Victoria the same across deceased-offender relationship type? 5. What are the key factors associated with homicide perpetrated by a family member against older adults in Victoria? 6. Are the key factors associated with homicide perpetrated by a family member against older adults in Victoria the same as for younger adults?

### 1.6. Aims

This study will examine the epidemiology of homicides among community-dwelling older adults (65+ years) compared to younger adults (18‐64 years) in the Australian state of Victoria during the period 2000 to 2015, using data from the VHR. The specific aims are to: 1. Compare the annual frequency and rate per 100,000 population of homicide amongst community dwelling older and younger adults in Victoria;

2. Compare the individual, interpersonal, incident, and community level factors associated with specific older adult homicide typologies; and

3. Compare the individual, interpersonal, incident, and community level factors associated with family homicide among community-dwelling older and younger adults in Victoria identified from Aim 1.

## 2. Methods and analysis

This study was developed and reported in accordance with the Strengthening the Reporting of Observational Studies in Epidemiology (STROBE) Statement for cross-sectional studies [86].

### 2.1. Study design

The study will be a jurisdictional population-based retrospective analysis of consecutive homicides of community-dwelling older adults in Victoria, Australia for the period 2001 to 2015.The proposed study protocol to our knowledge, provides the first descriptive comparison of familial homicide between younger and older adults through the lens of the social-ecological model providing detail at the individual-, relationship-,incident- and community-level. It is also the first study to compare the rates for this specific time period and geographic location, contributing to the response to calls for more international empirical data in this area of research [7,50]. The study has institutional human research ethics committee approval and the initial data extraction has taken place. At the time of manuscript submission data had not been processed or coded beyond what exists following data collection and entry, and no analysis had taken place.

### 2.2. Ethics

The project was approved by the Victorian Department of Justice and Regulation Human Research Ethics Committee on 1 June 2017 (renewed 10 December 2020; JHREC Reference CF/20/17503), endorsed by the State Coroner on 18 April 2017 (renewed 30 October 2020; CCOV RC Reference 376) and the Monash University Human Research Ethics committee (Project 29051, registered 17 May 2021).

### 2.3. Setting

The research setting is Victoria, a state of Australia, that in 2016 had a population of 5,926,624, 16% of which (922,603) was aged 65 years and older [87]. In 2021, there were 16 homicide incidents in Victoria involving and older victim aged 55 years and over and the rate for this group was 0.9 per 100, 000 population [88]. Victoria has seen a yearly increase in offender incidents of crimes against the person, that includes homicide and serious assault, with 49,000 recorded incidents for the period ending March 2022, up from 43,380 in 2018 [89].

The study will include adult homicide deaths reported to a coroner in Victoria between 1 January 2001 (the first year for which VHR data is available) and 31 December 2015, where the coroner’s investigation has been finalised. The time period was selected because coroners’ investigations in homicide investigations can take some years to finalise—the coroner by legal convention waits until proceedings in other jurisdictions (such as criminal trials and appeals) are concluded before commencing the coronial investigation—and 2015 was determined to be the most recent year for which most or all homicide investigations will be finalised at data collection.

### 2.4. Data source

The primary data source for the study is the VHR, a purpose-built database developed and maintained by the Coroners Prevention Unit, CCOV. The VHR is a Microsoft Power Platform database comprising coded, structured and unstructured free text entered following a review of the information generated for the Coroners’ investigations and Judges’ sentencing remarks (where present) for homicide and suspected homicide deaths. The VHR contains 242 variables comprising a core and enhanced dataset coded from Victorian coronial records, specifically: police report of death to the Coroner; post-mortem medical and scientific reports (e.g., autopsy and toxicology reports); the coronial brief; the Coroners’ finding; sentencing remarks from any criminal proceedings; and family violence death review reports (if present). Cases classified as family violence are coded with the additional enhanced dataset that includes specific data on history of family violence perpetration or victimisation for the deceased and offender.

Specially trained project officers at the CCOV maintain the VHR, where entries undergo regular and rigorous quality assurance, specifically full and partial validity checking, and random full-record entry by a second coder, to confirm inter-coder reliability and resolve disparities through consensus agreement.

An advantage of the VHR is the broad inclusion criteria on initial screening to facilitate the inclusion of all potential homicides in the database, regardless of whether an offender is identified, which is updated once the investigation is completed by the Coroner (see Table 2 for detailed definitions). This reduces the likelihood of selection bias by ensuring homicide cases are included in the dataset, regardless of criminal conviction status, and that it is kept up-to-date as new information arises.

**Table 2:** Definitions of terms in this protocol.

	Definition
**Community-dwelling**	A community dweller is a person living either in a private or publicly owned dwelling, or homeless at the time of the incident, and not in an institution (for example a prison, hospital, aged care home or other residential care).
**Older adult**	An older adult is defined as a person aged 65 years and older as defined by the Australian Government and more broadly in Australia and other OECD countries, for example the UK and the US [90–92].
**Homicide**	Homicide is defined in ICD-10 Version:15 as ‘injuries inflicted by another person with intent to injure or kill, by any means’ that includes neglect and abandonment (www.icd.who.int/browse10/2015/en#/X85). For this study, we will adopt the definition used by the CCOV for the VHR that includes the ‘unlawful killing of a person, where the death occurred or was suspected to have occurred due to external causes attributable to a person through assaultive force’. This definition includes: murder, manslaughter, murder-suicides, infanticides and homicides as classified by the Victorian Police regardless of offender apprehension or conviction, and includes those where the offender was excused from criminal liability. This definition excludes: driving-related fatalities not proximal to a crime, industrial accidents, unless manslaughter charges are laid, lawful homicide (i.e. by police in the course of duty), and missing persons unless a murder or manslaughter charge was laid.

The initial case identification process for the VHR is rigorous in that it includes known and suspected homicides at the onset of police investigation. A comparative study found that the National Homicide Monitoring Program, which uses a similar case identification method, yielded a higher number of homicides over a 12 year period than the causes of death data from the Australian Bureau of Statistics [93]. Case identification for the VHR is also more robust than other international homicide research that use voluntary reporting of homicides. For example, Chu identified that supplemental homicide reports may be missing 8% of homicide cases [43].

### 2.5. Case identification

Cases will be identified using the VHR where the homicide incident occurred between 1 January 2001 and 31 December 2015, and the Coroners’ investigation has been completed by 31 July 2017. Cases will be identified by an experienced member of the Coroners Prevention Unit (JD) against the inclusion criteria using a query of the VHR and exported to a Microsoft Excel spreadsheet.

### 2.6. Eligibility criteria

While the inclusion age for older adult homicide research can vary between populations and geographic locations, the predominant cut-off is 65 years [11,57]. This also suits the Australian context as it is the general administrative cut-off used to describe older adults [90].

**Fig 2:**
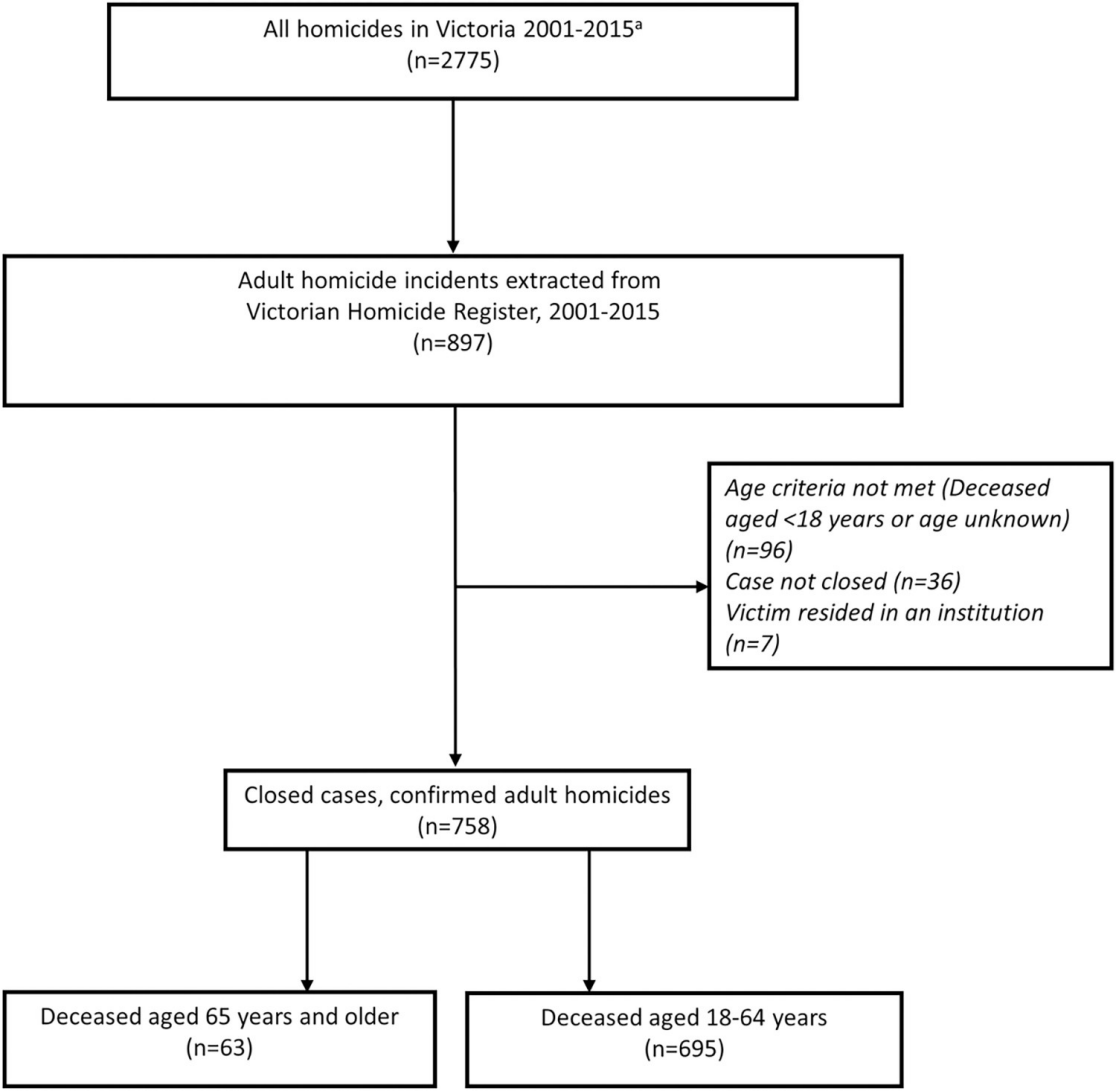
Case Identification diagram: Adult homicides in Victoria, Australia 2001–2015. ^a^Source: Victoria Police Crime Statistics.

Fig 2 shows the case identification process. Cases will be included if: the incident and death occurred in Victoria during study period of 1 January 2001 and 31 December 2015; the Coroners’ investigation and subsequent data entry is complete at time of data extraction; the deceased was aged 18 years or older at the time of the fatal incident; and the coroner determined that the death was the result of homicide.

Cases will be excluded if the incident occurred outside the Australian state of Victoria or the deceased was residing in an institution (e.g., nursing home, prison, mental health facility, hospital, detention centre) at the time of the incident.

### 2.7. Sample size

There are 758 eligible homicides for the study period, 63 older adult homicide victims (aged 65 years and older) and 695 younger adults (aged 18–64 years) to serve as a comparison group. Numbers will be verified during the data processing and descriptive analysis process. All of the eligible older adult homicides have been completely coded across the VHR domains relating to inclusion, sociodemographic, identity, context, mental illness, mental treatment and service contacts (described below and at Table 1). All of the younger adult homicides (n = 695) have been coded with core data suitable for summary comparison with older adult homicide victims (Table 1). This includes: age, sex, relationship, local government area (LGA) of incident, incident location type, mechanism of injury, use of excessive force (overkill) or presence of alcohol and drugs in the deceased and details of intimate partner relationship status, including intention to or recent separation and relationship duration.

### 2.8. Data collection/variables

The primary outcome measure for the study is the incidence of homicide in older adults. The secondary outcomes include the nature of older adult homicide (the individual-, interpersonal-, incident- and community-level factors described below); the nature of older adult homicide typologies based on the deceased-offender relationship (intimate and familial, acquaintance, stranger or unknown); and the comparison of older adult homicide with younger adult homicide. The variables to be extracted from the VHR for each homicide case of victims aged 65 years and older (and for a subset of younger adult victims) are shown in detail at Table 1, along with relevant research evidence and availability within the VHR for deceased, offender, older and younger adults.

Individual level variables include: demographic characteristics, substance use or exposure to violence; mental or physical illness diagnoses and treatment, and service contacts for both the deceased and offender (Table 1). The following new individual level variables will be generated from the existing data: Age group in ten-year brackets.

Interpersonal level variables include: the deceased-offender relationship, motive, and family violence history. New interpersonal level variables will include: Age difference between deceased and offender, motive (collapsed), and homicide-suicide.

Incident level variables include: location description (i.e. victim’s home), Overkill (excessive physical force), toxicology, injury mechanism, and justice outcomes. New incident level variables will include: season, day of week.

Community level variables includes LGA and new variables will include: Socioeconomic Index for Areas (SEIFA) Index of Relative Socioeconomic Disadvantage ranking and Australian Statistical Geography Standard remoteness structure for both deceased and offender residence and for the incident location.

### 2.9. Data de-identification

In accordance with coronial legislation, some of the information that this study seeks to examine includes records that state the identity of the deceased person. The records cannot therefore be accessed in a de-identified manner. The data extracted for this study will, however, be recorded in a de-identified manner. This will be done by removing any names, street addresses, day and month of birth, names of hospitals or other service providers, and free text fields used for annotation. Researchers will only access the VHR onsite at the CCOV and any data extracts will be stored without potentially identifiable information (as described above).

### 2.10. Data management

Data files will be accessed via password protected computers, and saved on secure drives at the Department of Forensic Medicine’s Southbank location according to Monash University and Victorian Institute of Forensic Medicine (which are co-located) IT security protocols.

Each data file will be password protected and only the researchers listed in this application will have access to the password(s). Investigators will work in adherence to Monash University’s Information Technology Policies and Procedures. Study data will be destroyed after a retention period of seven years.

### 2.11. Data analysis

All data analysis will be conducted using IBM SPSS Statistics for Windows, version 28.0 (IBM Corp., Armonk, N.Y., USA). The study is exploratory in nature so any findings regarding similarities or differences in the outcome measures are considered important. The importance of the results from chi-square and regression analyses will be determined by a p-value less than 0.05, and the magnitude and uncertainty in the estimated effect size.

#### 2.11.1. Description of older adult homicides

Descriptive statistics (numbers and percentages) will be generated for the homicide deceased and offenders (the primary outcome measure, Aim 1). Homicide rates and 95% confidence intervals will be calculated by year, sex and age bracket using denominator data from the Australian Bureau of Statistics [94], and compared as rate ratios using Poisson regression or negative binomial regression if the data exhibit overdispersion [95].

Descriptive statistics will be generated for older adult homicide deceased and their offenders for each of the variables outlined in Table 1 and cross-tabulated by sex (Aim 1).

For the secondary outcomes, older adult deceased and offenders will also be cross-tabulated by deceased-offender relationship (Intimate and familial, acquaintance, and stranger, Aim 2)and by age (older versus younger adults, Aim 3). Proportions for all secondary outcomes will be compared using Pearson’s chi-squared test (with Fisher’s exact tests where cell frequency assumptions are not met).

#### 2.11.2. Comparison of older and younger adult family homicide victims

If deemed feasible based on preliminary cross-tabulation, regression analysis will be used to compare all older adult homicide victims with all available younger adult homicide victim data where the offender was an intimate partner or family member (Aim 3). A selection of variables will be included in the binomial logistic regression, identified primarily as important through the research literature (see Table 1), and secondly through descriptive statistics (chi-squared test). Only variables demonstrating a statistically significant difference between the age groups will be included in the logistic regression.

Categorical variables will be recoded into binary variables for regression analysis by an experienced researcher (BK). All transformed and newly coded variables will be independently verified by a second researcher. Simple logistic regression and multiple regression analyses will be used to determine the association of variables described in Table 1 with homicide victim age status (older versus younger victim). Odds ratios with 95% confidence intervals will be used to estimate the relationship between selected variables and the outcome variable (65 years and older = 1) for inclusion in the multivariable model. The importance of an effect will be assessed by its p-value, the magnitude of its estimate, and the corresponding confidence interval. Sensitivity analysis will include stratifying age in both the younger and older adult groups (i.e., 65–74 years, 75–84 years and 85 years and older), year of incident, and separating homicides with a history of family violence.

A limitation of comparing older adult homicides with those of younger adults is that the younger reference group may be subject to variation within itself. For example, Fox and Levin [96] reported differences in race, robbery, motive and firearm use between younger homicide victims aged under 50 versus 50–64 years. Likewise, Addington [57] identified variability within their 18–64 age groupings for victim sex, relationship, location, motive and weapon. This will be controlled for in the proposed study through the division of both the older and younger groups into smaller (10-year) age categories and comparing these key variables to identify variability.

### 2.12. Bias

The initial screening process for the VHR is broad and so facilitates inclusion of all potential homicides and reduces the likelihood of selection bias because deaths with no known offender or without convictions can still be included.

The larger size of the reference group (aged 18–64) means that there will be differences in margins of error between the older and younger adult homicide groups. Due to the higher prevalence of homicide in the younger adult group, this is unavoidable.

### 2.13. Dissemination

Results from the analysis will be submitted for peer-reviewed publication, presented at conferences, and form part of BK’s PhD thesis. The publication of aggregated data will help practitioners, policy makers and other key stakeholders (including Coroners) that are concerned with the health and injury prevention of older Victorians, to make decisions to prevent future intentional‐cause deaths in older Victorians. Study findings will also be disseminated via relevant media networks on publication to ensure a greater audience for the research beyond the scientific community.

## 3. Conclusion

This study will be the first to describe older adult homicides using data from the VHR, a unique, high-quality and detailed data source describing homicide incidents, precipitating circumstances, and outcomes, including information on the deceased, offender, and their relationship. Older adult homicide will be explored using a social-ecological lens, contributing to an improved understanding of an under-developed field.

The study outcomes will benefit researchers and practitioners working in the prevention of violence and concerned with the health and safety of older adults living in the community. It will contribute high quality, reliable data and expanded the currently limited empirical research in the area. This is vital at a time of increased worldwide population ageing, elevated reported elder abuse, and little change to older adult homicide rate. The proposed study also includes a substantial study period as well as a comparison group.

By exploring older adult homicide typologies, and comparing their characteristics with those of younger adults, we can gain a clearer picture of how we can prevent future fatal violence in this growing and vulnerable cohort. While generalisability of the study outcomes is restricted by locality, the method affords good coverage of homicide that is more likely to be representative than for non-fatal abuse or assault data, and will provide a benchmark for measuring the impact of the Covid-19 pandemic.
